# Unveiling alpha-mannosidosis in Iraqi children: A series of clinically and genetically characterized cases with novel *MAN2B1* variant

**DOI:** 10.1016/j.ymgmr.2025.101282

**Published:** 2026-01-09

**Authors:** Mays Riyadh Al Tai, Nebal Waill Saadi, Marwa Sabah Alothman, Ikhlas Ali Ahmed, Hala Sameh Arif, Saja Baheer Abdulwahhab

**Affiliations:** a Children Teaching Hospital, Al Emammayn Al Khadimayn Medical City, Baghdad, Iraq; b College of Medicine, University of Baghdad, Paediatric Neurology unit - Children Teaching Hospital, Baghdad, Iraq; c Department of Pediatrics - College of Medicine, Al Nahrain University, Baghdad, Iraq; d Sidra Medicine, Doha, Qatar

**Keywords:** Alpha-mannosidosis, Lysosomal storage disorder, *MAN2B1* gene, Iraq, Diagnostic delay

## Abstract

**Background:**

Alpha-mannosidosis is a rare lysosomal storage disorder caused by *MAN2B1* mutations, leading to cognitive decline, hearing loss, infections, and skeletal abnormalities. Limited data exist from the Middle East; this study describes the clinical and genetic features of affected Iraqi children.

**Patients and methods:**

This study was conducted at Children Welfare Teaching Hospital, and Al Emamayn Al Khadimiyan Medical City Baghdad, Iraq. We retrospectively reviewed children diagnosed with alpha-mannosidosis (2017–2025). Diagnosis was confirmed by enzyme assay and *MAN2B1* testing. Clinical and imaging data were collected from medical records.

**Results:**

A total of nine children from five unrelated families were identified. The cohort included seven males and two females. The mean age at symptoms onset was 1.1 ± 0.5 years, while the mean age at diagnosis was 10.7 ± 7.6 years, indicating a diagnostic delay of approximately 9.6 ± 7.4 years. All the patients were born to consanguineous parents. The most common clinical features included psychomotor delay, sensorineural hearing loss and coarse facial features (100 % for each). Neuroimaging of the brain revealed variable findings, and skeletal radiographs showed dysostosis multiplex in 4/9 patients. Genetic testing revealed three pathogenic/likely pathogenic *MAN2B1* variants, including one novel variant [c.830C > T (p.Pro277Leu)].

**Conclusion:**

Our findings represent the first clinical and molecular characterization of alpha-mannosidosis in Iraqi children and reveal previously unreported genetic features in this population. It highlights that clinical and laboratory findings in our patients were largely consistent with previously published regional and international data. It demonstrates notable diagnostic delay and identified novel variants, expanding the mutational spectrum associated with the disease.

## 1 Introduction

Alpha-mannosidosis is a multisystem lysosomal storage disease inherited in an autosomal recessive manner, resulting from deficient activity of alpha-mannosidase. In humans, the disease is broadly characterized by facial and skeletal abnormalities, hearing impairment, intellectual disability and immune deficiency [1,2]. While the disease was originally classified into three broad clinical phenotypes (mild, moderate and severe), newer literature has suggested that patients are better placed within a continuum of symptoms and progression [3]. Alpha-mannosidosis is an ultra-rare condition. The disease has a reported prevalence of 1/1000000 people [4]. Initial screening typically involves detecting mannose-rich oligosaccharides in urine, while confirmation relies on demonstrating reduced acid alpha-mannosidase activity in leukocytes or fibroblasts [[5], [6], [7]]. Genetic analysis of the *MAN2B1* should be used to confirm the enzymatic diagnosis and may be used for prenatal diagnosis and genetic counselling of family members, but it should not replace biochemical testing [6]. Early diagnosis would allow for faster treatment initiation to avoid the full manifestations of often life-threatening symptoms. Although alpha-mannosidosis has been well-documented in some regions, it remains vastly under-recognized in many low- and middle-income countries, particularly in the Arabic countries including those from Middle East [[8], [9], [10], [11]]. In such settings, the disorder is frequently misdiagnosed or unrecognized due to its phenotypic overlap with more common neurodevelopmental and syndromic conditions, a lack of disease awareness, and limited access to enzymatic or genetic testing. Furthermore, high rates of consanguineous marriage [12] in the region may increase the prevalence of autosomal recessive disorders, including alpha-mannosidosis, yet population-specific data are scarce. In Iraq, no published studies to date have systematically described the clinical or molecular features of alpha-mannosidosis. This study aims to fill this critical knowledge gap by presenting the first detailed phenotypic and genotypic analysis of a cohort of Iraqi children diagnosed with the disease. By characterizing the clinical presentation, radiologic findings, and molecular variants observed in this population, we seek to increase recognition of alpha-mannosidosis in the region and emphasize the need for improved diagnostic access and targeted clinical care.

## 2 Patients and methods

### 2.1 Study design and participants

This cross sectional study was conducted at Children Welfare Teaching Hospital (CWTH) and Al Emamayn Al Khadimiyan Medical City, Baghdad, Iraq (two of the biggest tertiary centers in Iraq), from January to September 2025. It included a total of nine patients diagnosed with alpha-mannosidosis. Data for four patients who were diagnosed with alpha-mannosidosis during the period from 2017 to 2025, were retrospectively collected from medical records at CWTH. Five additional patients were referred from Al Emamayn Al Khadimiyan Medical City - metabolic unit and were enrolled prospectively during the study period and evaluated thoroughly. Patients were identified based on enzymatic analysis and molecular genetic testing for mutations in the *MAN2B1*.

### 2.2 Inclusion criteria

Patients diagnosed with alpha-mannosidosis based on biochemical testing (low alpha-mannosidase enzyme activity) and molecular genetic confirmation of pathogenic *MAN2B1* mutations.

### 2.3 Data collection

Data collected including the following variables:

• Demographics: Current age, age at onset of initial symptoms, age at diagnosis, sex, parental consanguinity, and family history of metabolic or genetic disorders.
• Clinical features: Detailed assessment of presenting symptoms, including developmental delay, motor and cognitive impairments, hearing loss, dysmorphism (coarse facial features), skeletal abnormalities, frequency of infections, and disease severity. Signs and symptoms of the disease were noted. Follow–up duration was calculated. The termination point for each patient in this study was defined as no further follow-up or death.
• Laboratory findings: Results of biochemical tests, including alpha-mannosidase enzyme activity in dried blood samples (DBS).
• Genetic analysis
• Radiologic findings: Imaging studies, including brain CT / MRI and X-rays of the skeleton.
• Audiological assessment: to detect hearing impairment.

### 2.4 Genetic testing

Four patients underwent genetic testing at the Centogene laboratory in Germany, including gene sequencing in one patient and a Centometabolic panel in three patients. Centogene laboratory is certified by the College of American Pathologists, the Clinical Laboratory Improvement Amendments (CLIA), and the International Organization for Standardization basis. While five patients were tested in the Archimedlife Laboratory (Vienna, Austria), which is certified by the European Molecular Quality Network (EMQN), the International Organization for Standardization (ISO), and accredited according to national standards.

Briefly, the *MAN2B1* in our patients was analyzed using next-generation sequencing (NGS) approaches: 1) Amplicon-based NGS covering all coding exons and conserved exon–intron junctions (one patient, Centogene); 2) Targeted NGS panels (CentoMetabolic® MOx) with enrichment of coding regions, 10 bp flanking intronic sequences, and known pathogenic variants, followed by alignment to hg19, variant calling, and classification according to ACMG guidelines (three patients, Centogene). Biochemical assays were performed when relevant variants were detected; 3) TruSight One (TSO) Illumina panel sequencing of *MAN2B1* in four patients (Archmidis Lab).

The genetic variants in our patients were classified according to established guidelines. One laboratory (CENTOGENE) applied the ACMG/AMP/ClinGen SVI recommendations, classifying the variants as pathogenic, likely pathogenic, or of uncertain significance. The other laboratory described the variants following HGVS nomenclature guidelines (2016 update) for standardized reporting of sequence variants.

### 2.5 Ethical considerations

The study adhered to the ethical principles outlined in the Declaration of Helsinki. Ethical approval for the study was obtained from the Institutional Review Board (IRB: No. 16, 1st of November 2024) of CWTH, and parental consent was obtained for the inclusion of patients. Data were anonymised to maintain confidentiality and stored securely.

### 2.6 Statistical analysis

Descriptive statistics were used to summarize demographic and clinical characteristics. Continuous variables, such as age at diagnosis, were expressed as mean ± SD, depending on data distribution. Categorical variables, such as sex, family history, and clinical features, were expressed as frequencies and percentages.

## 3 Results

A total of nine (out of five unrelated families) patients diagnosed with alpha mannosidosis were included in the study. Patients' demographics and clinical profiles for this case series are shown in Table 1.

**Table 1:** Demographic and clinical characteristics of children with alpha mannosidosis.

Variables	P1	P2	P3	P4	P5	P6	P7	P8	P9
Sex	F	M	M	M	M	M	M	M	F
Current status and age	Deceased at age 5 years	Alive 20 years	Alive 8 years	Alive 15 years	Alive 2 years 10 months	Alive 1 year 9 months	Alive 10 years 10 months	Alive 17 years	Alive 23 years
Age of onset (mo.)	10 mo.	18 mo.	18 mo.	24 mo.	6 mo.	6 mo.	12 mo.	12 mo.	12 mo.
Age at Dx (yr.)	1 year	19 years	7 years	14 years	2.67 years	1.6 years	10.8 years	17 years	23 years
Diagnostic delay (yr.)	0.2 year	17.5 years	5.5 years	12 years	2.2 years	1.1 years	9.8 years	16 years	22 years
Follow up time	4.6 years	1 year	1 year	1 year	^#^Pending follow up	^#^Pending follow up	^#^Pending follow up	^#^Pending follow up	^#^Pending follow up
Consanguinity Family history	+ -	+ +	+ +	+ -	+ +	+ +	+ +	+ +	+ +
First signs and symptoms	Hypotonia HI	DD Course facial features	DD	DD HI	Abdominal distension HSM*	Abdominal distension HSM*	DD	DD	DD
Psychomotor delay	No motor or speech development	Independent walking at a delayed age, currently ambulatory, non-verbal, comprehend and obey simple commands	Independent walking at a delayed age, currently ambulatory, non-verbal, comprehend and obey simple commands	Independent walking at a delayed age, currently ambulate with minimal assistance, non-verbal, comprehend and obey simple commands	Sat at 10 months, walked at 20 months, speaks 4 words, feeds self with a spoon	Sat at 7 months, currently cruising, speaks 3 words, feeds self with a spoon	Walked at 3 years, currently unsteady gait, absent speech	Walked at 4 years, unsteady gait, wheelchair-bound by 8 years, absent speech	Walked at 4 years, absent speech, currently wheelchair-bound by 15 year
Dysmorphism	Y	Y	Y	Y	Y	Y	Y	Y	Y
Skeletal abnormalities	Y	Y	Y	Y	N	Y	Y	Y	Y
Hearing impairment	Y (severe)	Y (moderate)	Y (moderate)	Y (severe)	Y (unknown severity)	Y (unknown severity)	NA	NA	NA
Ocular	Normal	Normal	Normal	Normal	Normal	Proptosis/papilledema	Normal	Normal	Normal
Organomegally	N	N	N	N	N	Current examination just palpable spleen and liver 4 cm below costal margin	N	N	N
Neurological	Generalized hypotonia, dystonia, weakness of limbs	Normal	Normal	Dystonia	Normal	Normal	Ataxia hypertonia, hyperreflexia	Dystonia hypotonia (axial)	Dystonia hypotonia (axial)
Psychiatric/Behavioral disturbances	N	N	Aggressive behavior	Aggressive behavior	N	N	N	Aggressive behavior	N
Frequent infections	(URTI)	(URTI)	(URTI)	(URTI)	Pneumonia Diarrhea	Pneumonia Diarrhea	(URTI)	(URTI)	(URTI)
Disease severity	Severe	Moderate	Moderate	Moderate	Moderate	Moderate	Moderate	Moderate	Moderate

Abbreviations: DD, developmental delay; F, female; HI, hearing impairment; HSM, hepatosplenomegaly; M, male; mo., month; N, no; NA, not available; ND, not done; P, patient; URTI, upper respiratory tract infections; Y, yes; yr., year. The patients' relatedness is reported in P2/P3, P5/P6 and P7/P8/P9. * Hepatosplenomegaly (HSM) was documented in two patients (P5 and P6) during infancy based on medical records from another hospital. In our current evaluation, HSM was only evident in P6 who had palpable liver and spleen on examination. +: sign is present, −: sign is absent. ^#^Patients seen recently; no follow-up data yet.

**Patients' demographics** revealed that seven were males, with a male-to-female ratio 3.5:1.The mean age at diagnosis was 10.7 ± 7.6 years, with a range from 1 to 23 years. The mean age of onset of clinical symptoms was 1.1 ± 0.5 years, with a range from 6 months to 2 years. The mean time to diagnosis after onset of symptoms was 9.6 ± 7.4 years. All patients had consanguineous parents, and one patient had a potentially relevant family history; a sister was deaf and paternal–side cousins who had dysmorphisms and were deaf and paternal uncles and aunts who had motor disability (deceased). The median follow-up period was 14.8 (0.4–92.2) months. Till the time of writing the report, eight patients are still alive and one patient has deceased.

**Clinical characteristics** showed that two thirds (6/9) of the patients included in this case series manifested features of alpha–mannosidosis during the first year of life. Psychomotor delay, dysmorphology, recurrent infections and hearing impairment were the most consistently reported signs and symptoms (100 % for each). Skeletal abnormalities were found in 8/9 (88.9 %) and hypotonia was found in three patients.

**Fig. 1:**
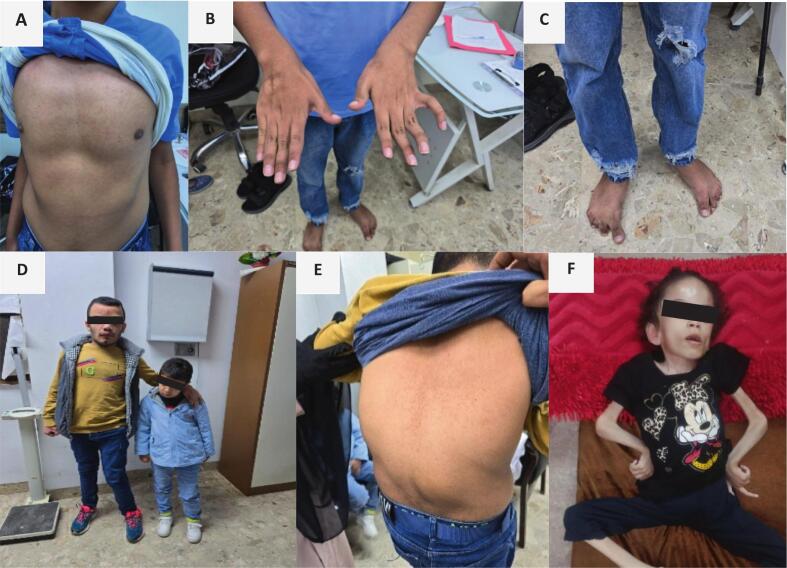
(A–F): Skeletal deformities and dysmorphism in children with alpha-mannosidosis. A–C (P4): shield chest and mild carinatum deformity; B and C: claw hands, swine neck and foot deformity (dystonia); D and E (P2 and P3 siblings): dysmorphic facies (prominent forehead, depressed nasal bridge, prognathism) and genu valgum (older sibling) and macrocephaly and limited neck extension (younger sibling) and E, kyphotic deformity in the older sibling; F (P1): facial dysmorphology, severe muscle wasting, and joint deformities in the upper limbs especially hands with posturing (dystonia).

Distinct skeletal deformities and craniofacial dysmorphologies characteristic of alpha-mannosidosis are illustrated in Fig. 1.

**Radiological and laboratory findings** were demonstrated in Table 2. Neuroimaging (CT and MRI of brain) revealed abnormalities in 4/6 (66.7 %) patients and 2/6 (33.3 %) with normal imaging. Dysostosis multiplex was found in 4/9 (44.4 %) patients. Audiological testing confirmed sensorineural hearing loss in all tested patients. Laboratory testing showed a significant reduction in alpha-mannosidase enzyme activity in five patients. Genetic testing revealed three different mutations in *MAN2B1* gene. Four patient shared the same novel mutation, which have not been previously reported in other populations.

**Table 2:** Laboratory, imaging and molecular findings in children with alph-mannosidosis.

Variables	P1	P2	P3	P4	P5	P6	P7	P8	P9
X-ray	Dysostosis multiplex	Lumbar Scoliosis	Dysostosis multiplex	N	Dysostosis multiplex	Dysostosis multiplex	N	N	N
Abdominal US	Normal	Normal	Normal	Normal	NA	NA	Normal	Normal	Normal
Echo	Normal	Normal	Normal	Normal	NA	Normal	Normal	Normal	Normal
IgG	NA	Normal	Normal	Normal	Low	Normal	NA	NA	NA
Brain imaging	Brain CT normal	Brain CT (12 years) calvarial bone thickening, dilated ventricles and hyperdensities in the pulvinar area of the thalami	Brain MRI (3 years) VRS (in the DWM)	Brain MRI normal	NA	NA	Brain MRI PVWM T2/FLAIR hyperintensity & Cerebellar atrophy	NA	Brain MRI PVWM/DWM T2/FLAIR hyperintensity & Cerebellar atrophy
Genotype	c.2436 + 1G > A (p.Glu786_Met812del27)	c.418C > T (p.Arg140*)	c.418C > T (p.Arg140*)	c.830C > T (p.Pro277Leu)	c.418C > T (p.Arg140*)	c.418C > T (p.Arg140*)	c.830C > T (p.Pro277Leu)	c.830C > T (p.Pro277Leu)	c.830C > T (p.Pro277Leu)
	Intronic splice site	Non-sense	Non-sense	Missense	Non-sense	Non-sense	Missense	Missense	Missense
	Likely pathogenic	Pathogenic	Pathogenic	Likely Pathogenic	Pathogenic	Pathogenic	Likely Pathogenic	Likely Pathogenic	Likely Pathogenic
	Homozygous	Homozygous	Homozygous	Homozygous	Homozygous	Homozygous	Homozygous	Homozygous	Homozygous
Alpha manosidase activity	11.0 μmol/l/h (≥16.2 μmol/l/h)	10.7 μmol/l/h (≥16.2 μmol/l/h)	11.4 μmol/l/h (≥16.2 μmol/l/h)	0.2 μmol/l/h (≥2.2 μmol/l/h)	1.1 μmol/l/h (≥2.2 μmol/l/h)	1 μmol/l/h (≥2.2 μmol/l/h)	0.6 μmol/l (≥2.2 μmol/l/h)	0.8 μmol/l (≥2.2 μmol/l/h)	0.9 μmol/l (≥2.2 μmol/l/h)

Abbreviations: CT, computed tomography; DWM, deep white matter; Echo, Echogardiography; F, female; IgG, Immunoglobuline G; M, male; MRI, magnatic resonance imaging; mo., month; N, no; NA, not available; ND, not done; P, patient; PVWM, periventricular white matter; US, ultrasound; VRS, vercew Robin spaces; Y, yes; yr., year. The patients' relatedness is reported in P2/P3, P5/P6 and P7/P8/P9 patients.

**Fig. 2:**
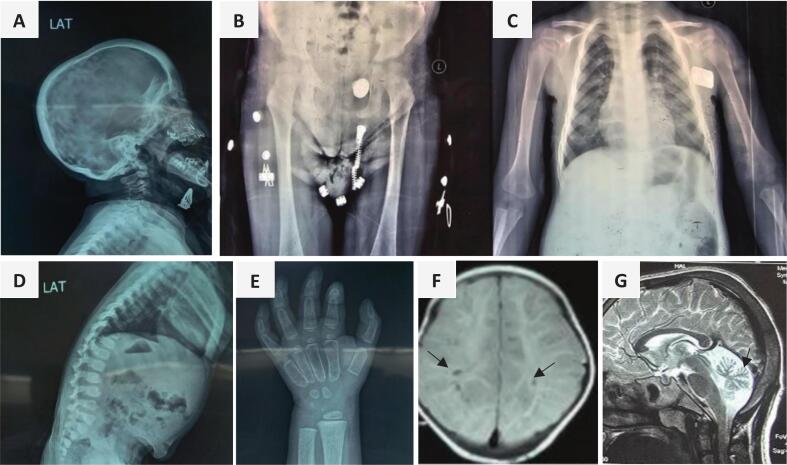
(A–G): Imaging findings in patients with alpha-mannosidosis: A–E, (Dysostosis multiplex): A) P5 - macrocephaly and calvarial thickening (beaten copper is also manifested); B) P4 - Underdeveloped acetabulum with rounded iliac crest; C) P4 - posteriorly widened ribs; D) P5 - ovoid (anterior beaking) vertebral bodies and gibbus; E) P5 - hand shows bullet-shaped phalanges; F) P4 - MRI findings (T1WI), Prominent Virchow – Robin spaces (black arrows); G) P9 - MRI brain, (T2WI sagittal), cerebellar atrophy.

The combined skeletal and neuroimaging findings characteristic of alpha-mannosidosis are shown in Fig. 2.

**Comparative analysis with regional and international cohorts:** To contextualize our findings, we compared the clinical and molecular characteristics of our cohort with those reported in regional and international studies. The data from the present Iraqi cohort (*n* = 9) are presented alongside cohorts from the UAE, Tunisia, Turkey, and Italy, as shown in Table 3. It reveals both commonalities and distinct differences in the clinical and radiological presentation of alpha-mannosidosis.

**Table 3:** Comparison of patients' characteristics and radiological findings in our cohort and published regional/international studies.

Features	Present study (2025) *n* = 9	UAE study (2025) *n* = 9^[^^8^^]^	Tunisian study (2021) *n* = 7^[^^9^^]^	Turkish study (2024) *n* = 16^[^^10^^]^	Italian study (2024) *n* = 9^[^^14^^]^
Sex	M:F 3.5:1	M:F 3:1	F:M 2.5:1	M:F 1.67:1	M:F 2:1
Age of onset (year)	1.1 ± 0.5	0.33 ± 0.6	ND	1.8 ± 0.9	ND
Age of diagnosis (year)	10.7 ± 7.6	10 ± 11.8	12.5	6.6 ± 4.7	ND
Diagnostic delay (year)	9.6 ± 7.4	10.5 ± 10.7	ND	4.6 ± 4.3	11.8
Psychomotor delay/regression	100 %	ND*	6/7 (86 %)	3 (18 %)	100 %
Facial dysmorphism	100 %	7/9 (78 %)	7/7 (100 %)	94 %	100 %
Hearing impairment	100 %	8/9 (89 %)	4/7 (57 %)	94 %	100 %
Recurrent infections	100 %	7/9 (78 %)	3/7 (43 %)	14 (87 %)	8/9 (89 %)
Skeletal deformities	89 %	5/9 (56 %)	7/7 (100 %)	10 (62 %)	9/9 (100 %)
Organomegaly	22 %	3/7 (43 %)	4/7 (57 %)	6/13 (46 %)	Most
X-ray changes	56 %	4^⁎^	7/7 (100 %)	ND	ND
Abnormal Brain Imaging	67 %	3/4 (75 %)	1^⁎^	5/10 (50 %)	100 %

Abbreviations: F, female; M, male; n, number; ND, not determind. (*) denote values extracted from text where applicable, not from clearly defined denominators.

## 4 Discussion

This case series presents the first clinically and genetically characterized cohort of children with alpha-mannosidosis from Iraq. Our findings underscore the classical phenotype of this rare lysosomal storage disorder, including developmental delay, coarse facial features, hearing impairment, and recurrent respiratory infections. Uncovering deleterious variants in *MAN2B1* gene in all patients, including potentially novel mutation, adds to the expanding global mutational spectrum and highlights the diagnostic value of molecular testing, particularly in consanguineous populations.

In this study, we report the clinical characteristics and molecular analysis of nine Iraqi patients from five unrelated families managed in specialist centers in Baghdad. At the time of writing this report, the nine cases were the only ones diagnosed in Iraq. In 2019 *MAN2B1* gene sequencing diagnosed the first patient after the MPSs enzyme panel test came out negative. Following the implementation of the Centometabolic panel into clinical practice at Children Welfare Teaching Hospital in December 2023 and continuing through May 2024, the following three cases were diagnosed. At May, and then September 2025 additional five siblings were diagnosed at Al Emamayn Al Khadimiyan Medical City based on the MPS phenotype. MPSs panel enzyme testing was performed in five patients; however, when the findings were negative, a gene sequencing and Centometabolic panel were used to diagnose mannosidosis. The neurologist and the metabolic specialist in charge of the P4 and P7/P8/P9 patients, respectively, care had a high clinical index of suspicion, therefore, the patients were promptly genetically tested for mannosidosis. Diagnosing eight patients within 21 months likely reflects growing clinical awareness and improved diagnostic efforts, which have helped uncover previously unrecognized cases in a highly consanguineous population.

The comparison of our cohort with regional (UAE, Tunisia, Turkey) [[8], [9], [10]] and international (Italy) [13] studies is revealed in Table 3 which highlights that psychomotor delay/regression, facial dysmorphism, hearing impairment, and recurrent infections are the most consistent features across studies, often approaching 100 %, reinforcing their value as hallmark features. Skeletal deformities are also frequent, though less uniform, while organomegaly appears less common in the present cohort, which might reflect differences in disease severity. A considerable diagnostic delay, averaging nearly a decade, remains a prominent challenge across all series. This is justified by ultra-rarity of the disease, and thus unfamiliarity of many clinicians with it, non-specificity of the early symptoms, phenotypic variability, slow progression of symptoms, limited access to diagnostic facilities (namely genetic testing in the local practice), clinical overlapping with other lysosomal storage disorders, and finally delay in referral to specialists. Turkish study [10] showed the shortest reported time to diagnosis which may be explained by combination of systemic healthcare strength, awareness and availability of diagnostic infrastructures.

Three patients had hypotonia. One (P1) was examined during infancy and hypotonia was documented. The other two (P8/P9) were assessed more recently by adult neurologists, and hypotonia was also noted at that time. For the remaining patients with infantile onset, hypotonia was not reported because they were not examined by the authors during infancy, and no detailed medical records from that early period were available.

All nine patients demonstrated global developmental delay of varying degrees. Motor milestones were uniformly delayed, with most patients achieving independent walking later than expected, and some eventually requiring assistance or becoming wheelchair-dependent. Speech development was severely affected, ranging from a few spoken words in mildly affected individuals to complete absence of speech in others. Despite expressive language deficits, several patients were able to comprehend and follow simple commands. Overall, the cohort exhibited significant and often progressive impairment in both motor and language domains, consistent with the neurodegenerative nature of alpha-mannosidosis.

In P1, enzyme activity was measured at nearly 60 % of normal in a non-fibroblast assay (DBS), yet this seemingly reassuring result contrasted sharply with the clinical reality of a severe, rapidly progressive course that ended in death at 5 years of age. In contrast, Patients 4, 7, 8, and 9 had a significantly lower enzyme level—around 10 % of normal—but showed a more moderate disease course with relatively slower progression. This apparent discrepancy should be interpreted cautiously, as DBS assays have inherent variability and are less reliable for assessing genotype - phenotype correlation compared to fibroblast or leukocyte enzyme testing. Nevertheless, such differences emphasize that enzyme activity alone does not fully explain disease severity in lysosomal storage disorders. Additional factors - including genetic modifiers, environmental influences, and immune-mediated mechanisms - may also modulate clinical expression, as has been shown in other lysosomal diseases [[14], [15], [16]]. Again, this could be attributed to the c.2436 + 1G > A mutation in P1, an intronic splice-site alteration that has been predicted to cause a more severe phenotype of the disease. In LSD patients, clinical variability is high even among patients with similar enzyme levels. This suggests that modifier genes, environmental factors, and possibly immune responses also influence severity. Therefore, enzyme levels may be predictive of broad disease category (mild vs. severe), but not precisely predictive of all clinical manifestations. In Gaucher disease, variants in the *SCARB2* or *PSAP* may modulate neurological involvement [14]. In Fabry disease, polymorphisms in *ACE* and *eNOS* have been linked to cardiovascular phenotype variability [15]. Similar variability in clinical presentation among siblings (with the same *MAN2B1* mutations) was reported [9]. Chronic inflammation can worsen neurological or systemic disease - acting as a positive feedback loop [16].

P1 presented with atypical features for classic alpha-mannosidosis, including severe muscle wasting, low birth weight, and pronounced dysphagia. The early and severe presentation of alpha-mannosidosis in this patient with the severe dysphagia might contribute to her clinical profile with the possibility of an additional or overlapping genetic or perinatal disorder. Unfortunately, due to financial limitations, the family could not afford whole exome sequencing to explore other potential genetic aetiologies. The diagnostic process in this case was step-wise: she was initially evaluated for mucopolysaccharidoses (MPSs) through enzyme assays and relevant genetic testing, which were negative. Subsequently, alpha-mannosidase enzyme activity in DBS was found to be reduced, and sequencing of the *MAN2B1* confirmed the diagnosis.

Our study is among the first in the Arab world to offer comprehensive genetic data, thereby enriching regional mutation databases and emphasizing the need for molecular confirmation in suspected cases. This study provides the first molecular insight into alpha-mannosidosis among Iraqi children, revealing both known and novel variants in the *MAN2B1*.

Among the variants detected, c.418C > T is detected in four patients (from two unrelated families) and was previously documented in international databases as pathogenic, confirming their clinical significance [10,17]. The patients shared several features, including early age of onset, coarse facial features (in three siblings), intellectual disability, developmental delay, and skeletal abnormalities - similar to the patient reported in the Turkish study [10]. One of the patients, who is 8 years old, did not exhibit coarse facial features as early as his sibling, reflecting intra-familial variability.

We identified a novel variant [c.830C > T (p.Pro277Leu)] that has not been reported in the literature or global variant databases. It was classified as likely pathogenic, with multiple in silico tools (PolyPhen-2, SIFT, MutationTaster) supporting its damaging effect, while gnomAD reported a very low allele frequency (0.000016). P4 carrying this variant showed moderate disease severity with severe, early-onset hearing loss requiring cochlear implantation at age 4, while his sister also had hearing impairment but tested negative on enzymatic assay, suggesting possible contribution of other genetic factors. Importantly, this variant was also identified in P7, P8, and P9, who, together with P4, all harbor the c.830C > T mutation. While they belong to two separate families, both families originate from the same province in Iraq, suggesting a potential founder effect.

Access to enzymatic assays and genetic testing in Iraq remains limited. Most patients in this study were diagnosed with the support of international laboratories (Archimed Lab and Centogene), as local facilities for both types of testing are currently unavailable.

Disease-modifying treatments are not accessible in Iraq. Enzyme replacement therapy is unavailable, while hematopoietic stem cell transplantation (HSCT) is neither approved nor performed locally for the indication of alpha-mannosidosis. This stark disparity underscores the global inequities in rare disease care and the need for broader access to emerging therapies. Management for our patients was largely supportive, including physical therapy, speech therapy, hearing aids, and symptomatic treatment for recurrent chest infections. Despite these interventions, the disease imposed a substantial burden: P8 and P9 became wheelchair dependent, P4 required assistance to walk, and P1 had severe psychomotor delay and eventually died at 5 years of age. Recurrent upper respiratory tract infections were universal, with several patients requiring repeated hospitalizations, adding to the significant strain on both patients and their families. HSCT has been used as a disease-modifying therapy for alpha-mannosidosis in multiple centres and series, with growing experience and improving safety profiles. Early series and case reports described variable outcomes, and larger multi-institutional cohorts followed. More recently, Šáhó et al. [18] reported outcomes in 21 children transplanted after 2010 and documented clinically meaningful improvements (hepatomegaly, recurrent infections, and partial improvement in hearing) and an improved safety profile with no deaths during follow-up in their cohort, although transplant-related complications (infections, acute and chronic GvHD) occurred. These data, taken together with prior reports, support that HSCT is an established therapeutic option for selected patients with alpha-mannosidosis, particularly when performed early in the disease course [19,20]. The absence of multidisciplinary care models and specialized centres for lysosomal storage disorders further restricts optimal long-term management.

### 4.1 Novelty and limitations

To the best of our knowledge, this is the first study to describe the clinical and molecular features of alpha-mannosidosis in an Iraqi cohort. The identification of novel *MAN2B1* mutation not previously reported in public databases adds to the global mutational spectrum of the disorder and highlights the importance of including underrepresented populations in genomic studies. It also identifies local actionable gaps in diagnosis and care. However, this study has certain limitations. The relatively small sample size (*n* = 9) limits the generalizability of the findings and reduces the power to establish definitive genotype–phenotype correlations. Given the rarity of alpha-mannosidosis, geographically restricted cohort, the results should be interpreted with caution. Larger, multicenter studies encompassing diverse regions of Iraq are warranted to validate and expand upon these observations. Long-term clinical and neurocognitive follow-up data were lacking for some patients. Furthermore, biochemical tests were incomplete, as leukocyte alpha-mannosidase activity (normalized to /mg protein) or demonstration of increased mannose in urine oligosaccharide analysis could not be tested.

## 5 Conclusion

Our findings represent the first clinical and molecular characterization of alpha-mannosidosis in Iraqi children and reveal previously unreported genetic features in this population, adding to the regional and global knowledge of this ultra-rare disorder. It highlights that clinical and laboratory findings in our patients were largely consistent with previously published regional and international data. It demonstrates notable diagnostic delay and identified novel variant, expanding the mutational spectrum associated with the disease. Improving early diagnosis in resource-limited settings requires increasing clinicians' awareness of early clinical signs, facilitating access to metabolic and genetic testing, and establishing referral networks to support timely management.

## CRediT authorship contribution statement

**Mays Riyadh Al Tai:** Writing – review & editing, Writing – original draft, Methodology, Formal analysis, Data curation, Conceptualization. **Nebal Waill Saadi:** Writing – review & editing, Writing – original draft, Methodology, Formal analysis, Data curation, Conceptualization. **Marwa Sabah Alothman:** Writing – review & editing, Data curation. **Ikhlas Ali Ahmed:** Writing – review & editing, Project administration. **Hala Sameh Arif:** Data curation. **Saja Baheer Abdulwahhab:** Data curation. **Fatma Al Jasmi:** Writing – review & editing, Writing – original draft, Methodology, Formal analysis, Data curation, Conceptualization.

## Funding

The authors declare no conflicts of interest related to the content of this manuscript.

## Declaration of competing interest

The authors declare that they have no known competing financial interests or personal relationships that could have appeared to influence the work reported in this paper

## Data Availability

The data that has been used is confidential.

## Appendix A Appendix

Cento metabolic panel

Genomic DNA is enzymatically fragmented, and regions of interest are enriched using DNA capture probes. The final indexed libraries are sequenced on an Illumina platform. For the CentoMetabolic® MOx panel, the coding regions, 10 bp of flanking intronic sequences, and known pathogenic/likely pathogenic variants (coding and non-coding) of the *ABCA1, ABCC2, ABCD1, ABCD4, ADA, AGA, AGL, AGPS, ALAD, ALAS2, ALDOA, ALDOB, ALPL, APOA2, APOA5, APOB, APOC2, APOE, ARG1, ARSA, ARSB, ASAH1, ASL, ASS1, ATP7A, ATP7B, BCKDHA, BCKDHB, CBS, CETP, CLN3, CLN5, CLN6, CLN8, CPOX, CPS1, CTNS, CTSA, CTSD, CTSK, CYP11B1, CYP17A1, CYP19A1, CYP21A2, DBT, DHCR7, ENO3, ENPP1, EPHX2, FECH, FGF23, FUCA1, G6PC, G6PD, GAA, GALC, GALE, GALK1, GALNS, GALT, GBA, GBE1, GHR, GK, GLA, GLB1, GM2A, GNPAT, GNPTAB, GNPTG, GNS, GUSB, GYG1, GYS2, HCFC1, HEXA, HEXB, HFE, HJV, HGD, HGSNAT, HMBS, HPRT1, HSD3B2, HYAL1, IDS, IDUA, ITIH4, KHK, LAMP2, LCAT, LDHA, LDLR, LIPA, LIPI, LMBRD1, LPA, LPL, MAN2B1, MANBA, MCOLN1, MFSD8, MMACHC, MMADHC, NAGA, NAGLU, NAGS, NEU1, NPC1, NPC2, OTC, PAH, PEX1, PEX10, PEX12, PEX13, PEX14, PEX16, PEX19, PEX2, PEX26, PEX3, PEX5, PEX6, PEX7, PFKM, PGAM2, PGK1, PGM1, PHKA1, PHKA2, PHKB, PHKG2, PKLR, POR, PPOX, PPP1R17, PPT1, PRKAG2, PYGL, PYGM, RBCK1, SGSH, SLC17A5, SLC25A13, SLC25A15, SLC25A36, SLC2A1, SLC2A2, SLC2A3, SLC3A1, SLC3A2, SLC40A1, SLC6A19, SLC7A7, SLC7A9, SLCO1B1, SLCO1B3, SMPD1, SUMF1, TFR2, TPP1, UGT1A1, UMPS, UROD, UROS, IVD, DLX4, ANTXR2, ABCB4, ABCG5, ABCG8, ACAT1, AGXT, ALDH4A1, ALG3, LDLRAP1, BTD, CD320, CPT1A, DDC, DIABLO, DNAJC5, DPYD, ETHE1, FAH, FBP1, GAMT, GATM, GYS1, HLCS, HPD, LIPC, MMAA, MMAB, MMUT, PCSK9, PDHB, PNPO, PSAP, SI, SLC22A5, SLC25A20, SLC37A4, SLC6A8, TAT genes are targeted for analysis.*
